# CRISPR/dCAS9-mediated DNA demethylation screen identifies functional epigenetic determinants of colorectal cancer

**DOI:** 10.1186/s13148-023-01546-1

**Published:** 2023-08-24

**Authors:** Juan Ramón Tejedor, Alfonso Peñarroya, Javier Gancedo-Verdejo, Pablo Santamarina-Ojeda, Raúl F. Pérez, Sara López-Tamargo, Ana Díez-Borge, Juan J. Alba-Linares, Nerea González-del-Rey, Rocío G. Urdinguio, Cristina Mangas, Annalisa Roberti, Virginia López, Teresa Morales-Ruiz, Rafael R. Ariza, Teresa Roldán-Arjona, Mónica Meijón, Luis Valledor, María Jesús Cañal, Daniel Fernández-Martínez, María Fernández-Hevia, Paula Jiménez-Fonseca, Luis J. García-Flórez, Agustín F. Fernández, Mario F. Fraga

**Affiliations:** 1 Nanomaterials and Nanotechnology Research Center (CINN), Spanish National Research Council (CSIC), 33940 El Entrego, Asturias, Spain; 2 Health Research Institute of the Principality of Asturias (ISPA), Avenida de Roma S/N, 33011 Oviedo, Asturias, Spain; 3 Spanish Biomedical Research Network in Rare Diseases (CIBERER), 28029 Madrid, Spain; 4 Institute of Oncology of Asturias (IUOPA), University of Oviedo, 33006 Oviedo, Asturias, Spain; 5 Viralgen Vector Core, 20009 San Sebastián, Gipuzkoa, Spain; 6 Department of Organisms and Systems Biology, Institute of Biotechnology of Asturias, University of Oviedo, 33071 Oviedo, Asturias, Spain; 7 Institute for Research in Biomedicine, The Barcelona Institute of Science and Technology, 08028 Barcelona, Spain; 8 Maimónides Biomedical Research Institute of Córdoba (IMIBIC), 14071 Córdoba, Spain; 9 Department of Genetics, University of Córdoba, 14071 Córdoba, Spain; 10 Division of General Surgery, Department of Colorectal Surgery, Central University Hospital of Asturias (HUCA), 33011 Oviedo, Asturias, Spain; 11 Division of Oncology, Department of Medical Oncology, Central University Hospital of Asturias (HUCA), 33011 Oviedo, Asturias, Spain; 12 Department of Surgery and Medical Surgical Specialties, University of Oviedo, 33006 Oviedo, Asturias, Spain

**Keywords:** DNA methylation, Gene expression, Epigenetics, Tumour suppressor gene, CRISPR screen, Colorectal cancer

## Abstract

**Background:**

Promoter hypermethylation of tumour suppressor genes is frequently observed during the malignant transformation of colorectal cancer (CRC). However, whether this epigenetic mechanism is functional in cancer or is a mere consequence of the carcinogenic process remains to be elucidated.

**Results:**

In this work, we performed an integrative multi-omic approach to identify gene candidates with strong correlations between DNA methylation and gene expression in human CRC samples and a set of 8 colon cancer cell lines. As a proof of concept, we combined recent CRISPR-Cas9 epigenome editing tools (dCas9-TET1, dCas9-TET-IM) with a customized arrayed gRNA library to modulate the DNA methylation status of 56 promoters previously linked with strong epigenetic repression in CRC, and we monitored the potential functional consequences of this DNA methylation loss by means of a high-content cell proliferation screen. Overall, the epigenetic modulation of most of these DNA methylated regions had a mild impact on the reactivation of gene expression and on the viability of cancer cells. Interestingly, we found that epigenetic reactivation of *RSPO2* in the tumour context was associated with a significant impairment in cell proliferation in p53^−/−^ cancer cell lines, and further validation with human samples demonstrated that the epigenetic silencing of *RSPO2* is a mid-late event in the adenoma to carcinoma sequence.

**Conclusions:**

These results highlight the potential role of DNA methylation as a driver mechanism of CRC and paves the way for the identification of novel therapeutic windows based on the epigenetic reactivation of certain tumour suppressor genes.

**Supplementary Information:**

The online version contains supplementary material available at 10.1186/s13148-023-01546-1.

## Background

Colorectal cancer (CRC) is a complex and multifactorial pathology in which both genetic and epigenetic alterations are crucial to its pathogenesis and its molecular heterogeneity [1]. The epigenomic landscape of DNA methylation in CRC is altered during tumorigenesis, where local hypermethylation of promoter regions and global hypomethylation of DNA is frequently observed [2, 3]. From an historic perspective, it was widely assumed that aberrant hypermethylation of promoters in cancer was associated with the repression of gene expression [4]. Indeed, elegant approaches integrating transcriptomic and epigenomic data from cell lines obtained from CRC patients revealed the presence of DNA regions whose methylation status was associated with gene expression changes necessary for tumour growth [5, 6]. On the other hand, numerous studies have tried to demonstrate, both at the gene level and at the whole genome level, a causal relationship between methylation changes and the regulation of gene expression through the use of DNA demethylating reagents [7]. Although these works have identified numerous associations between DNA methylation and gene expression changes, these relationships did not fully confirm the driving functional role of DNA methylation changes in tumour development, since demethylating drugs have considerable pleiotropic effects [8].

The lack of molecular tools that allow the ectopic regulation of DNA methylation levels in specific regions of the genome has been an experimental limitation for decades. Therefore, distinguishing the DNA methylation changes actually involved in the carcinogenic process from the rest of the transiently accumulating epigenetic aberrations represented a major challenge in the field. The discovery of the gene editing technology mediated by Clustered Regularly Interspaced Short Palindromic Repeats (CRISPR) [9] has opened up a field for the study of numerous physiological and pathological processes, including cancer. This technology allows the introduction of “on demand” genetic insults to a certain region of the genome through the action of the Cas9 nuclease in complex with specific guide RNAs in a highly specific and precise manner. Interestingly, recent experimental evidence has demonstrated that the combination of a catalytically inactive unit of the Cas9 nuclease (dCas9) with different catalytic domains of effector proteins can serve as a powerful tool for the transcriptional and epigenetic edition of certain regions of the genome [10–12]. Of note, the use of these technologies has unravelled the impact of DNA methylation in multiple cellular processes including cell differentiation [13], cancer [14] and neurodevelopmental disorders [15, 16].

In this work, we have combined the use of computational resources with recent state-of-the-art epigenome editing approaches to identify potential epigenetic drivers of CRC. We first identified a set of candidate genes with strong correlations between DNA methylation and gene expression in different human CRC cohorts and a set of 8 colon cancer cell lines. To determine the functional consequences exerted by these inferred epigenetic aberrations, we modulated the DNA methylation status of 56 promoters displaying strong epigenetic repression in CRC using a customized arrayed gRNA library and the CRISPR-dCas9 epigenome and transcriptional editing toolbox (dCas9-TET1, dCas9-TET-IM, dCas9-VP64), and the potential functional consequences of this epigenetic reactivation were addressed by means of a high-content cell viability screen of DLD1 and HCT116 cells. Interestingly, we found that epigenetic reactivation of *RSPO2* in the tumour context was associated with a significant impairment in cell viability in DLD1 and HCT116 cancer cell lines. An additional exploration of data from familial adenomatous polyposis (FAP) and CRC patients indicated that the epigenetic silencing of *RSPO2* is a progressive event that maximizes at mid-late stages in the adenoma to carcinoma sequence. These observations confirm the potential role of DNA hypermethylation as a driver mechanism of CRC and may facilitate the identification of novel therapeutic targets related to the progression of the tumour.

## Results

### Identification of DNA methylation alterations in colorectal cancer

**Fig. 1:**
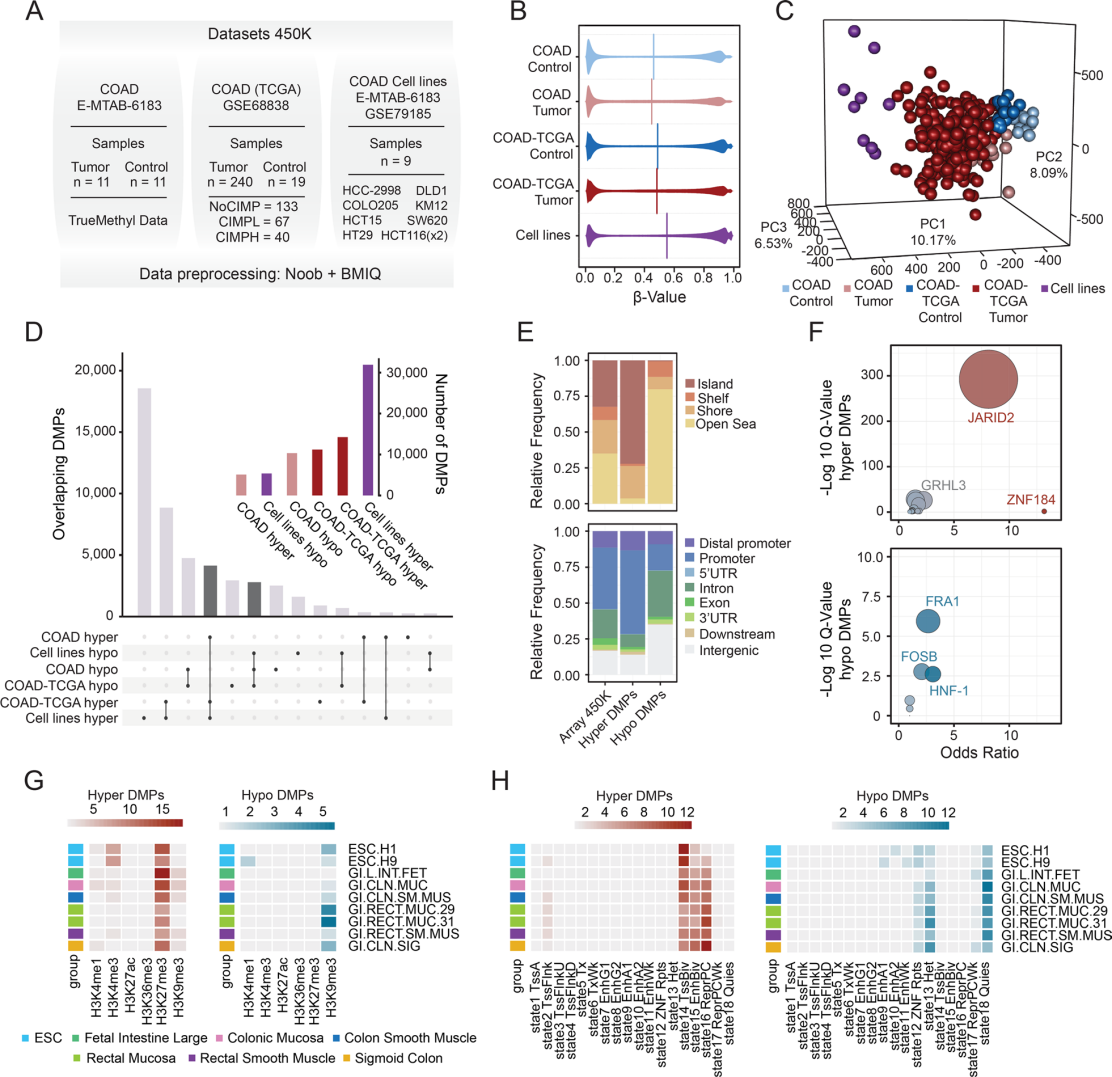
The DNA methylation landscape of CRC samples and cell lines. **A** Table indicating the datasets used in this study for the identification of CRC specific DMPs. **B** Violin plots depicting the overall 5mC estimates from the 450 K platform in control and CRC samples. Vertical lines indicate the median value for each of the above-mentioned distributions. **C** Principal component analysis for 413,756 CpG sites across all samples included in the DNA methylation study. Samples are coloured according to their clinical status (control/tumour) in their corresponding dataset. **D** Barplot indicating the number of common (dark-grey) and specific significantly hyper- or hypomethylated CpG sites as compared with healthy controls observed in the comparisons indicated (FDR < 0.05, mean β difference > 0.25). The inset illustrates the total number of hyper- and hypomethylated CpG sites observed in each separate condition. **E** Stacked barplots displaying the relative frequency of significant common hyper- or hypomethylated CpGs in relation to their CpG context (top) or CpG location (bottom). The background distribution of the 450 K platform is included for interpretation purposes. **F** Bubble plots showing enrichment of TFBS in the context of common hyper- (top) or hypomethylated (bottom) CpG sites as determined by the information obtained from the GTRD database. Dot size denotes statistical significance (−log10 adjusted *p* value) of a particular TFBS dataset as compared with the background distribution of the 450 K platform. **G** Heatmaps illustrating histone mark enrichment analyses of common hyper- and hypomethylated CpGs. Colour scales represent the odds ratio obtained across 6 common histone modifications from the NIH Roadmap Epigenome consortium as compared with the background distribution of the 450 K platform. The legend indicates the tissue types used for these comparisons. **H** Same as G, but displaying chromatin state enrichment analyses across 18 chromatin states obtained from the NIH Roadmap Epigenome consortium

To depict the overall DNA methylation landscape of CRC samples, we analysed a set of 11 colon tumours and paired healthy colon mucosa samples, obtained from the Central University Hospital of Asturias, using the TrueMethyl protocol on the high-content Infinium 450 K array platform. In addition, we performed a simultaneous analysis of 240 colon adenocarcinoma samples and 19 healthy controls from the publicly available TCGA repository (GSE68838) [17] and a panel of 8 CRC cell lines obtained from the NCI60 cell line resource (GSE79185) [18] with the aim of characterizing robust DNA methylation changes arising in different patient cohorts (Fig. 1A, Additional file 1: Table S1). CRC samples were characterized by a global significantly lower DNA methylation status than in healthy colon mucosa, while CRC cell lines displayed, on average, higher DNA methylation levels than the primary samples from patients (Fig. 1B). As expected, an unsupervised principal component analysis (PCA) segregated CRC methylomes from healthy colon mucosa and CRC cell lines (Fig. 1C). CRC cell lines accumulated the vast majority of significant differentially methylated probes (DMPs) as compared to healthy colon mucosa, with 31,899 hyper- and 5,355 hypomethylated DMPs (Fig. 1D, Additional file 2: Table S2). In the context of the CRC patients, we identified 5,102 hyper- and 10,332 hypomethylated DMPs in our local dataset, while a total of 14,248 hyper- and 11,207 hypomethylated DMPs were observed for the CRC samples from the TCGA consortia.

To focus on those aberrant DNA methylation changes present in multiple CRC cohorts, as well as in CRC cell lines, which is an experimental system more amenable for epigenetic-editing interventions, we focused on those common alterations present in all datasets analysed. We found a total of 4,152 hyper- and 2,798 hypomethylated DMPs shared across our local dataset, the TCGA-COAD cohort and the panel of CRC cell lines (Fig. 1D). A detailed inspection of the genomic distribution of these common DMPs revealed an enrichment of hypermethylated CpGs at CpG islands and promoter regions (Fisher’s tests *p* < 0.001, OR, respectively, 5.49 and 1.87), while hypomethylated CpGs were enriched in open sea locations and intergenic regions (Fisher's tests *p* < 0.001, OR, respectively, 7.47 and 2.69) (Fig. 1E). We next performed a transcription factor binding site (TFBS) enrichment analysis of these common DMPs using the gene transcription regulation database (GTRD) [19]. These analyses revealed a significant differential enrichment of hypermethylated CpG sites in regions decorated with Polycomb2 complex, represented by the Jumonji/ARID Domain-Containing Protein 2 (JARID2) motif. In contrast, hypomethylated CpGs displayed a significant association with the AP-1 family members FRA1 and FOS, as well as a significant enrichment in binding sites of the hepatocyte nuclear factor-1 (HNF1A) (Fig. 1F and Additional file 3: Table S3).

To reveal the potential epigenomic impact of these common aberrant DNA methylation changes observed in CRC samples, we also performed a comprehensive region set enrichment analysis by using 6 publicly available histone datasets (H3K4me1, H3K4me3, H3K27ac, H3K27me3, H3K36me3, H3K9me3) comprising a total of 7 reference colorectal-related epigenomes and 2 embryonic stem cells from the Roadmap and ENCODE epigenome consortia [20, 21]. We found a differential enrichment between hyper- and hypomethylated CpGs, this methylation gain being associated with locations decorated with the polycomb-related H3K27me3 mark in all tissues analysed, and a significant enrichment in the H3K4me3 mark in embryonic stem cells (Fig. 1G, left, and Additional file 4: Table S4). On the other hand, the loss of DNA methylation at the common hypomethylated CpG sites was related to the constitutive heterochromatin-related H3K9me3 mark in all tissues analysed (Fig. 1G, right). In addition, we performed an enrichment analysis based on chromatin segmentation data from the same tissues, which also revealed that the functional distribution of commonly hyper- or hypomethylated CpG sites was substantially different (Fig. 1H and Additional file 5: Table S5). Hypermethylated CpG sites were enriched at genomic locations associated with flanking and bivalent transcription start sites (TSSs), bivalent enhancers and polycomb-repressed elements, while hypomethylated CpGs were mainly enriched at heterochromatin/quiescent regions and zinc finger repeats. Collectively, these results indicate that the aberrant DNA methylation observed in these independent cohorts seems to be functionally associated with different molecular mechanisms involved in the epigenetic remodelling of CRC.

### Integration of DNA methylation and gene expression data reveals potential functional epigenetic alterations in CRC samples

**Fig. 2:**
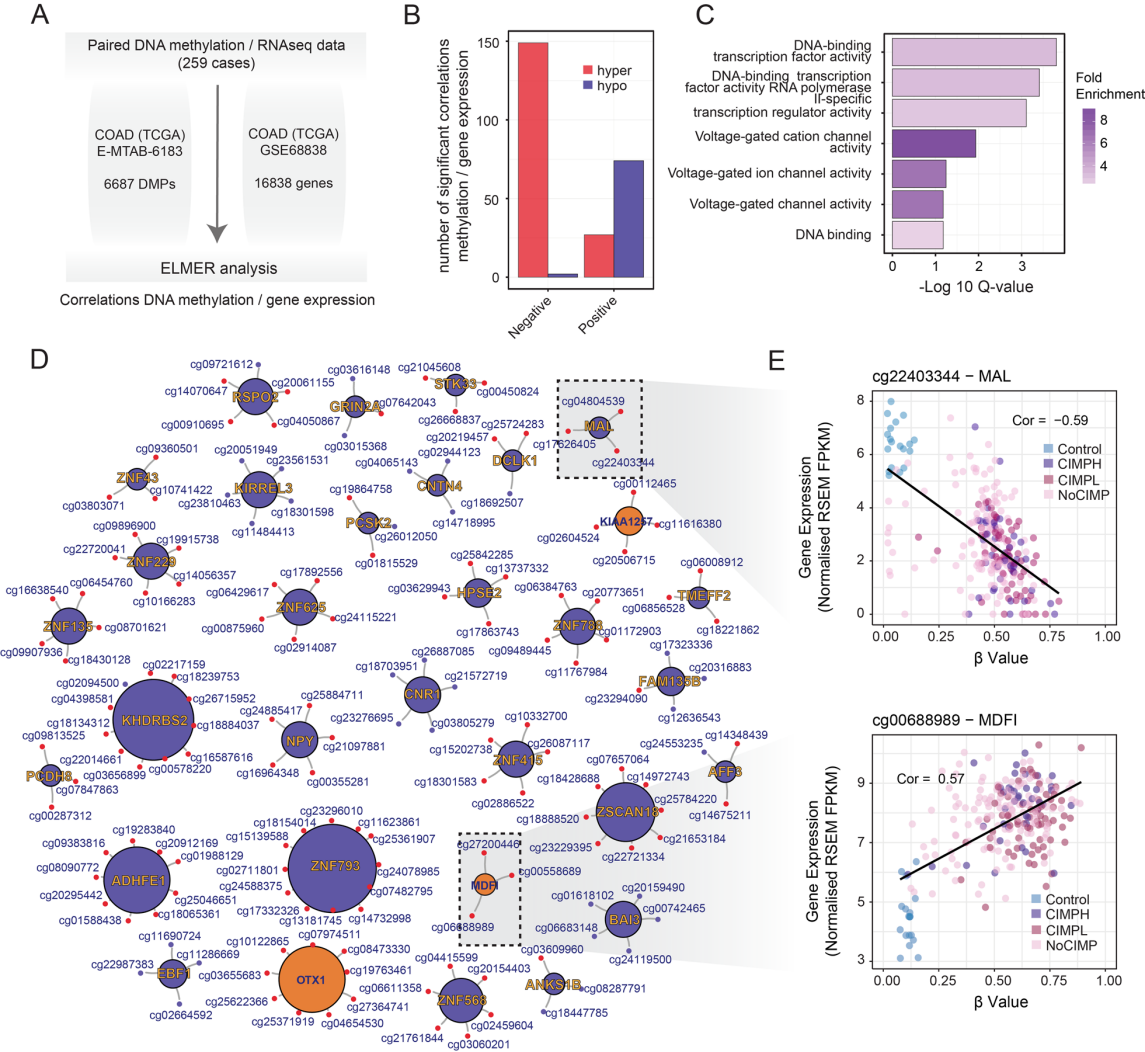
Inferring functional epigenetic alterations through integration of DNA methylation and gene expression data. **A** Schema depicting the integration of DNA methylation and RNA-Seq data using the ELMER algorithm. Values indicate the number of significant common hyper- or hypomethylated CpGs used in the context of the 450 K arrays and the number of genes expressed in the TCGA-COAD RNA-seq dataset. **B** Barplot illustrating the number of significant gene expression–correlating hyper- or hypomethylated CpGs associated with promoter regions with an absolute Pearson’s correlation > 0.5. **C** Barplot displaying gene ontology enrichment analyses of the significant gene expression–correlating hyper- or hypomethylated CpGs. Genes with a consistent correlation with DNA methylation were used for enrichment calculation versus the background dataset (16,838). Colour range denotes the odds ratio of the represented ontology, while bar size represents the significance of these enrichments (−Log10 adj. *p* value) as calculated with the GORILLA tool. **D** Graph illustrating the gene-CpG promoter network associated with significant gene expression–correlating hyper- (red) or hypomethylated (blue) CpGs in CRC samples. Genes that are down- or upregulated in CRC samples cells as compared to healthy controls are shown in blue or orange, respectively. **E** Scatter plots displaying the correlations between DNA methylation and gene expression for the genes *MAL* (top) and *MDFI* (bottom). Control and CRC samples are coloured according to their CIMP status, and the resulting significant correlation (*p* value < 0.001) is indicated for each gene comparison

With the aim of identifying those functional DMPs with a significant impact in the expression of nearby genes, we used ELMER [22] to integrate paired DNA methylation and RNA-Seq data in samples from the TCGA-COAD cohort. Robust correlations were calculated using those common DMPs identified in the different cohorts analysed (4,152 hyper- and 2,798 hypomethylated CpGs), and all the genes expressed in the RNA-Seq (Fig. 2A). We found that DNA hypermethylation correlated well with gene repression (151 negative gene-CpG pair correlations: 149 hyper- and 2 hypomethylated CpGs), while DNA hypomethylation changes were more associated with gene activation (101 positive gene-CpG pair correlations: 27 hyper- and 74 hypomethylated CpGs) (Fig. 2B and Additional file 6: Table S6). Gene expression–correlating DMPs were enriched at cellular pathways related to DNA binding, transcription factor activity and the regulation of transcription (Fig. 2C). As anticipated by the previous correlation analyses, we found that the top hypermethylated CpGs lead to gene repression (Fig. 2D), for instance hypermethylation of the CpG probe cg22403344 in CRC, located on the promoter region of the gene *MAL*, correlated with decreased expression of its target gene (Fig. 2E). In contrast, hypermethylation of CpGs located at gene bodies, such as in the case of the CpG probe cg00688989, was significantly associated with activation of the *MDFI* gene in CRC samples (Fig. 2E). These data confirm the potential functional role of a set of aberrant DNA methylated sites observed in clinical samples and they define a list of target genes with potential implications for the development of CRC.

### Modulation of DNA methylation levels using CRISPR-dCas9-mediated epigenome editing technologies

**Fig. 3:**
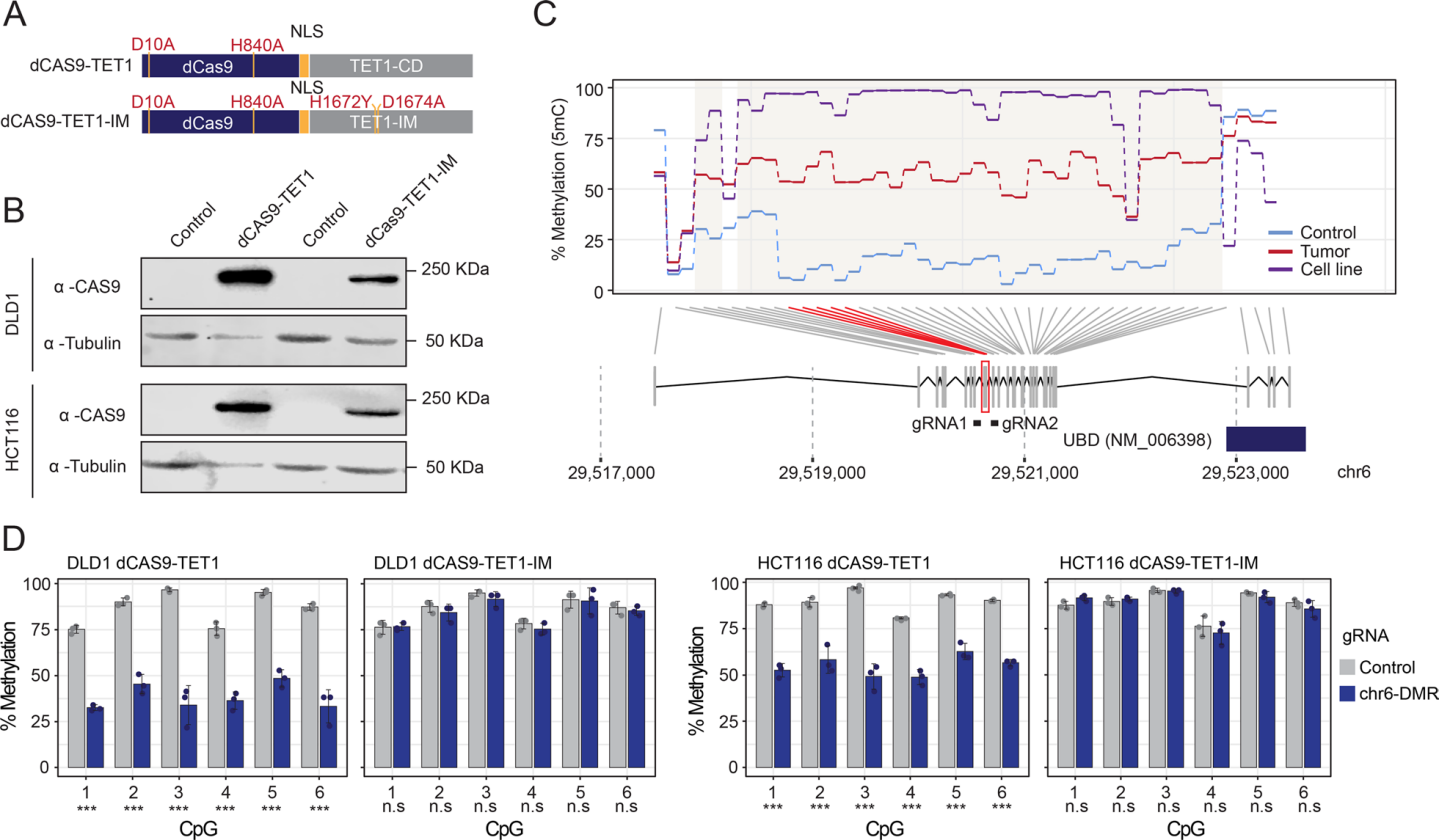
dCas9-TET1 induces locus-dependent DNA demethylation in DLD1 and HCT116 cells. **A** Schema illustrating the structure of the chimeric CRISPR-dCas9 construct fused to the catalytic domain (TET1, top) or the catalytically inactive domain (TET1-IM, bottom) of TET1. The position of the nuclear localization signal (NLS) and the mutations of the dCas9 or the TET1 catalytic domain are indicated. **B** Expression levels of chimeric dCas9-TET1, dCas9-TET1-IM and β-Tubulin proteins obtained by western blot analyses in control transduced or Cas9 transduced DLD1 and HCT116 cells. The approximate size of the protein products is indicated. **C** Line plot illustrating overall 5mC levels observed for a robust cancer-associated differentially hypermethylated region in control and tumour samples and in CRC cell lines. Data represent the average methylation status of the indicated CpG sites for the aforementioned categories. Significantly hypermethylated CpG sites observed in the differential methylation comparisons are highlighted in red. The genomic position of the gRNAs designed to modulate the DNA methylation status of this region is indicated. **D** Barplots representing the percentage of DNA methylation observed for the CpG sites included in the modulated differentially methylated region in DLD1 and HCT116 cells in the context of control gRNA (grey) or gRNAs targeting this DMR (blue) in cells transduced with dCas9-TET1- or dCas9-TET1-IM-related chimeras. Data represent mean ± standard deviation of at least 3 independent experiments, and two-sided Welch’s t tests were applied for the different statistical comparisons versus each corresponding control condition. ***p value < 0.001; n.s.—nonsignificant

Recent advances in the field of genomics have led to the development of state-of-the-art epigenomic-editing approaches based on the use of CRISPR/Cas9 technology [13]. To further explore the functional role of the observed promoter hypermethylation in CRC samples, we set up an epigenetic-editing strategy based on the expression of chimeric proteins in which the catalytic domain of the methylcytosine dioxygenase TET1 is fused to a dead Cas9 nuclease, thus permitting the specific in-vivo targeting of this complex at a certain genomic locus in the presence of their corresponding guide RNAs. Discrimination of steric from catalytic effect is achieved with the use of an additional chimeric construct that involves inactivating mutations in the catalytic domain of TET1 but preserving the size and the structure of the protein (Fig. 3A). Viral transduction of these chimeric constructs in DLD1 and HCT116 cells resulted in the stable expression of these epigenomic-editing tools in CRC cell lines (Fig. 3B). To test the efficacy of this method in the context of CRC cell lines, we first performed an additional in silico analysis to identify robust differentially methylated regions (DMRs) common to the three cohorts analysed in this study (local, TCGA-COAD and cell lines). We identified a total of 96 DMRs, distributed in 88 hyper- and 8 hypomethylated regions (Additional file 7: Table S7). To validate the efficacy of these epigenetic-editing tools, we designed two gRNAs against a robust DMR region that included more than 40 hypermethylated CpGs in Ubiquitin D (UBD) identified in all three cohorts analysed (Fig. 3C, Additional file 8: Fig. S1A). Epigenetic editing of this DMR using the dCas9-TET1 fusion protein led to a significant decrease (30–50%) of DNA methylation at this locus in both DLD1 and HCT116 cells (Fig. 3D), but the chimeric construct dCas9-TET1IM did not display any significant alteration in the DNA methylation status of this region, indicating that the catalytic activity of TET1 was mediating the DNA demethylation of this robust DMR. Of note, a detailed colony bisulphite sequencing experiment revealed that the area of influence of the dCas9-TET1 chimera spans 50 to 150 bp from the gRNA target sequence (Additional file 8: Fig. S1B), suggesting that the editing of DNA methylation is precise and specific to the genomic region of interest.

### Systematic functional interrogation of gene promoters subjected to DNA hypermethylation in CRC

**Fig. 4:**
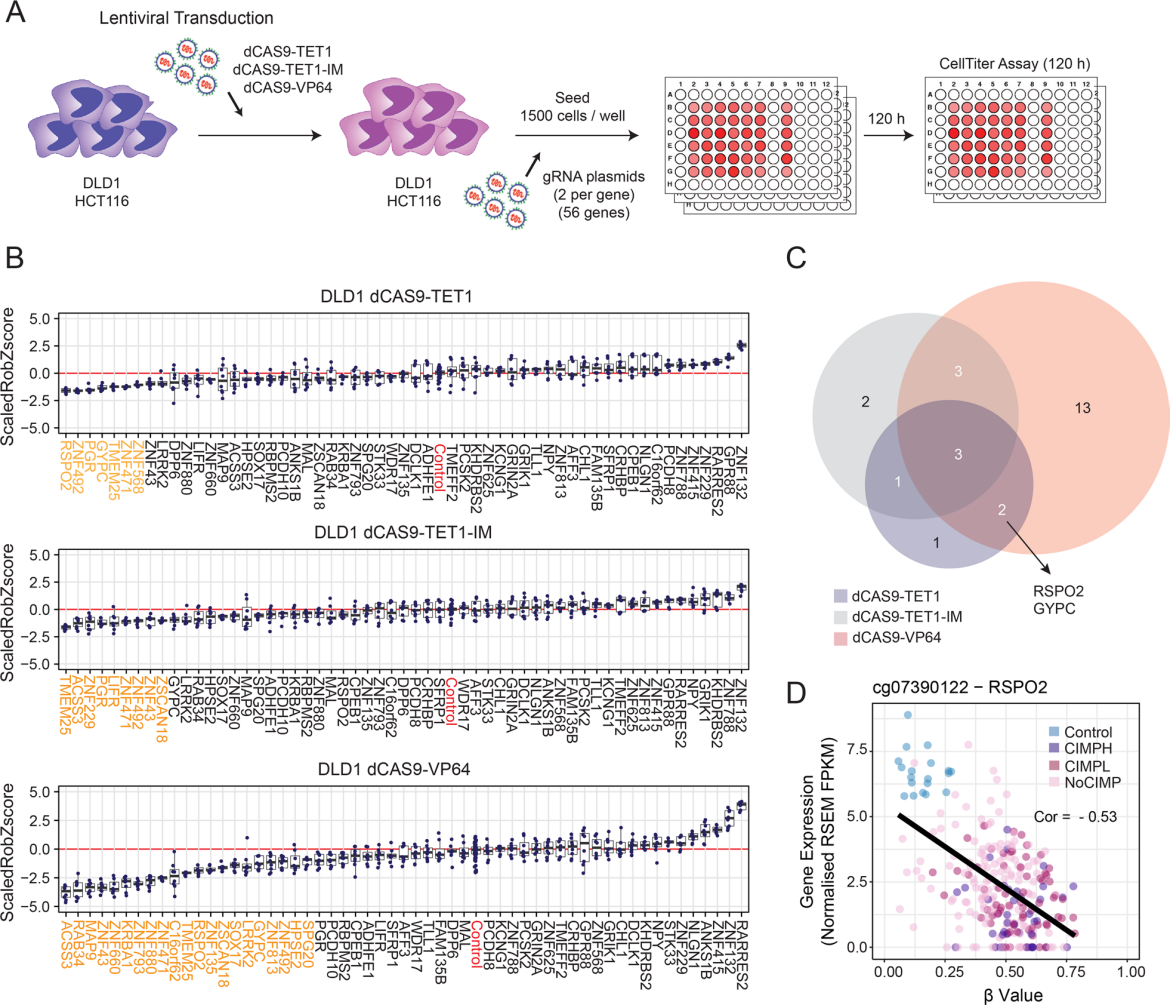
CRISPR-dCas9 demethylation screen identifies functional epigenetic drivers in DLD1 cells. **A** Schema depicting the experimental CRISPR-dCas9 mediated pipeline adopted in the screen strategy. A total of 56 gene promoters were targeted with 2 gRNAs each against genomic regions with significant DNA hypermethylation levels in tumour cells. The screen was performed in parallel using DLD1 cells transduced with the chimeric constructs dCas9-TET1, dCas9-TET1-IM or the transcriptional activator dCas9-VP64. **B** Boxplots displaying the Scaled Robust Z-score data observed for the indicated gene promoters in the context of DLD1 cells transduced with dCas9-TET1 (top), dCas9-TET1-IM (middle) or dCas9-VP64 (bottom) constructs. Those epigenetic modulations that resulted in statistically significant changes in the proliferation rate of DLD1 cells are highlighted in orange, while conditions corresponding to control gRNAs are highlighted in red. **C** Venn diagrams illustrating the overlap of significant hits obtained in the different screen strategies. **D** Scatter plots showing the correlation between DNA methylation and gene expression levels for the gene *RSPO2*

To assess the functional impact of promoter hypermethylation in CRC, we performed a high-content cell proliferation screen in DLD1 and HCT116 cells upon a targeted DNA demethylation of those 149 epigenomic regions with significant anti correlations with the expression of their nearby genes (56 in total). The catalytic activity of the TET1 dioxygenase was directed towards the hypermethylated promoters of these genes using the dCas9-TET1 chimeric construct, and 2 different lentiviral gRNAs per target gene were co-transduced to improve the efficiency of the epigenetic-editing system (Additional file 9: Table S8). To discern between steric versus catalytic effects and to control for effects mediated by transcriptional activation, we performed a simultaneous screen using the catalytically inactive domain of TET1 (dCas9-TET1-IM) and the dCas9-VP64 constructs respectively (Fig. 4A). Considering these combinations, a total of 1,488 conditions were analysed in a high-throughput cell proliferation approach using the MTT method (Additional file 10: Table S9). We found that 7, 9 and 21 genes significantly impaired cell proliferation of DLD1 cells in the context of, respectively, the dCas9-TET1, dCas9-TET1-IM and dCas9-VP64 conditions (Fig. 4B and 4C). The epigenetic modulation of 12, 6 and 10 genes significantly altered the cell proliferation status of HCT116 cells in the respective above-mentioned conditions (Additional file 11: Fig. S2A and S2B). Interestingly, targeted DNA demethylation of the promoter regions of *RSPO2* and *GYPC* genes exerted a significant effect in cell proliferation in DLD1 and HCT116 cells (Fig. 4C and Additional file 11: Fig. S2B), in terms of a catalytic effect mediated by dCas9-TET1 as well as transcriptional activation mediated by dCas9-VP64, but not through a potential steric mechanism, which was ruled out using the dCas9-TET1-IM construct. We validated a robust negative correlation between the expression levels of *RSPO2* and its DNA methylation status at its promoter region in all CRC samples analysed (Fig. 4D), suggesting that the impairment in cell proliferation may be the consequence of the epigenetic reactivation and subsequent restoration of the gene expression levels of *RSPO2* in CRC cancer cells.

### R-spondin-2 is epigenetically repressed in CRC along the adenoma to carcinoma sequence.

**Fig. 5:**
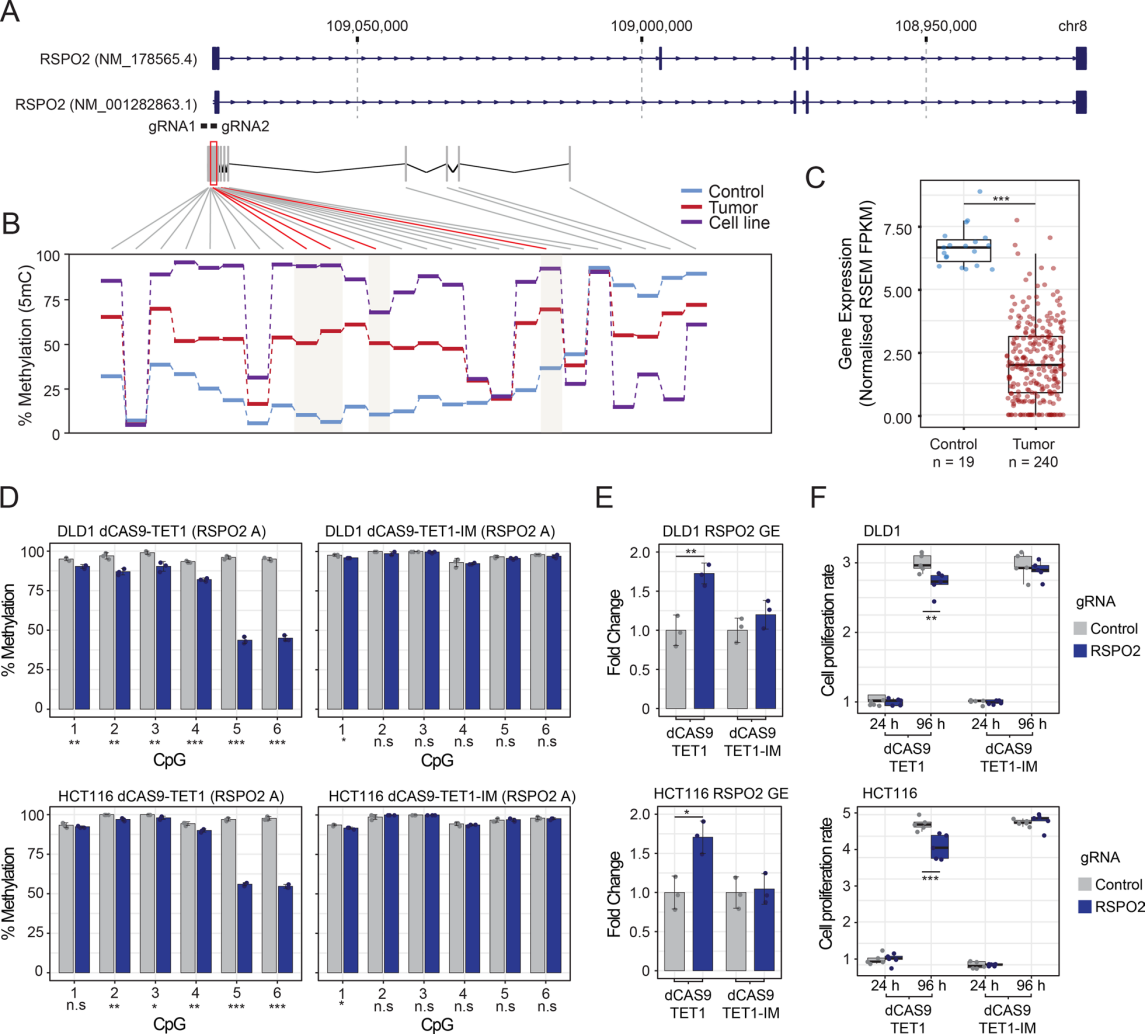
Epigenetic modulation of *RSPO2* impairs the proliferation rate of CRC cell lines. **A** Schema reflecting the genomic position of the *RSPO2* gene, the CpG sites analysed in the 450 K methylation platform and the gRNAs designed to modulate the DNA methylation status of its promoter region. **B** Line plot illustrating the average methylation status of the CpG sites for the indicated categories. Significantly hypermethylated CpG sites observed in the differential methylation comparisons are highlighted in red. **C** Boxplot showing the gene expression levels of the *RSPO2* gene in control or tumour cases obtained from the TCGA-COAD dataset. **D** Barplots depicting the percentage of DNA methylation observed for the CpG sites included in the *RSPO2* promoter region in DLD1 (top) and HCT116 (bottom) cells in the context of control gRNA (grey) or gRNAs targeting this modulated region in cells transduced with dCas9-TET1 (left)- or dCas9-TET1-IM (right)-related chimeras. **E** Barplots indicating *RSPO2* gene expression levels observed upon epigenetic modulation of its promoter region in DLD1 (top) and HCT116 (bottom) cells in the context of dCas9-TET1- or dCas9-TET1-IM-related chimeras, both in control and *RSPO2* targeting RNA conditions. **F** Boxplots displaying the normalized cell proliferation rate observed for the indicated gRNA treatments at two different time points (24 and 96 h) in the context of DLD1 and HCT116 cells transduced with dCas9-TET1 and dCas9-TET1-IM constructs. For D and E, data represent mean ± standard deviation of at least 3 independent experiments, while for F, at least 8 experimental replicas were included. Two-sided Welch’s *t*-tests were applied for the different statistical comparisons versus each corresponding control condition. ***p value < 0.001; **p value < 0.01; *p value < 0.05; n.s.—nonsignificant

A detailed inspection of the *RSPO2* promoter region revealed its consistent hypermethylation in CRC samples and CRC cell lines as compared to control tissue (Fig. 5A and 5B) in addition to the significant DMPs identified in the DNA methylation analysis (cg04050867, cg20061155, cg14070647 and cg09970569, Additional file 12: Fig. S3A and S3B), while an opposite trend was observed in the context of CpGs located at the intragenic region (Fig. 5A and 5B). These results were in agreement with the reduction in *RSPO2* expression in CRC samples (Fig. 4D and Fig. 5C). Targeted DNA demethylation with the dCas9-TET1 construct achieved a significant reduction in the DNA methylation status of the targeted region in DLD1 and HCT116 cells (Fig. 5D, Additional file 12: Fig. S3A, Amplicon RSPO2-A) but not in the region located ~ 250 bp downstream (Additional file 12: Fig. S3A, Amplicon RSPO2-B, Fig. S3C), nor in the context of the dCas9-TET1-IM catalytically inactive mutant (Fig. 5D, Additional file 12: Fig. S3C). Under these same conditions, the epigenetic modulation strategy led to a significant increase in *RSPO2* gene expression levels in both cell lines, but only in the context of the dCas9-TET1 catalytically active construct (Fig. 5E, ~ 1.7 fold change), despite the extent of this epigenetic-mediated transcriptional reactivation being of modest magnitude as compared to the gene expression activation achieved in the context of the dCas9-VP64 condition (Additional file 13: Fig. S4A and S4B, 35 to 76 fold change in DLD1 and HCT116 cells, respectively). A simultaneous analysis of the cell viability of these conditions revealed a significant impairment of cell proliferation upon *RSPO2* reactivation in DLD1 and HCT116 cells, both in the context of epigenetic reactivation (Fig. 5F) and of transcriptional reactivation mediated by the dCas9-VP64 system (Additional file 13: Fig. S4C), confirming the functional role of this factor in the above-mentioned cell lines.

**Fig. 6:**
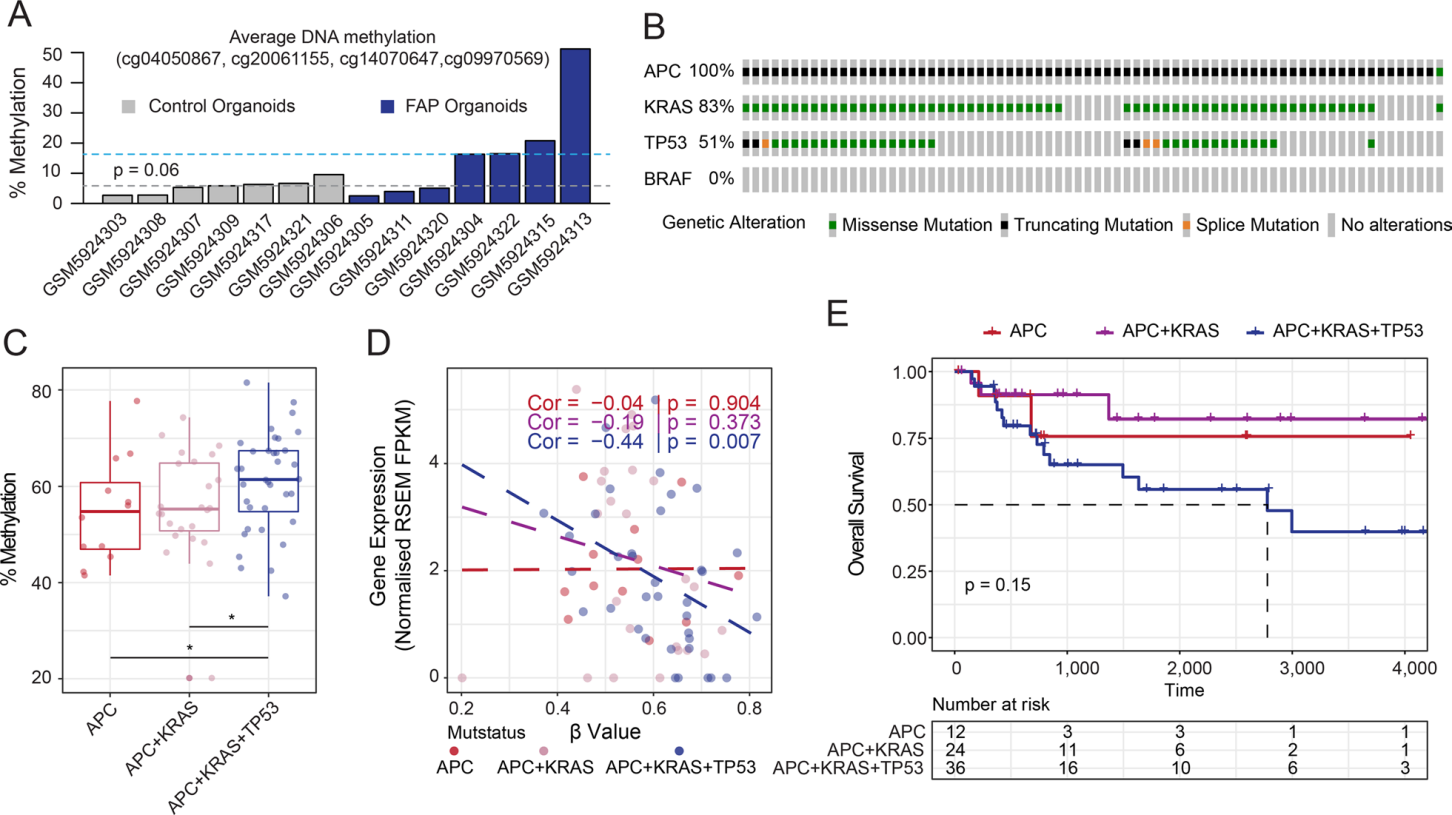
RSPO2 DNA methylation and gene expression levels are anti-correlated along the adenoma to carcinoma sequence. **A** Barplot depicting the DNA methylation status of colon organoids obtained from FAP or control patients. Data represent the average methylation value of the four CpG sites identified as differentially methylated in our study. Dashed lines indicate the median DNA methylation value of FAP or control organoids and statistical significance between these groups was calculated by means of a one-sided Welch’s *t*-test. **B** Oncoprint representation of the TCGA-COAD samples with mutations in the *APC*, *KRAS* and *TP53* genes included in these analyses. **C** Boxplots illustrating the DNA methylation score of the indicated samples along the adenoma to carcinoma sequence in the context of a single (*APC*), double (*APC* + *KRAS*) or triple mutant group (*APC* + *KRAS* + *TP53*). Statistical significance was inferred using a one-sided Wilcoxon rank sum exact test (*p value < 0.05). **D** Scatter plots showing the correlation between DNA methylation and gene expression levels for the gene *RSPO2* in the context of the above-mentioned categories. Resulting Spearman correlations and *p* values are indicated in the figure legend. **E** Kaplan–Meier plot showing the overall survival estimates of the single, double- and triple-mutant categories. *p* value refers to differences in event rates between the Kaplan–Meier curves and was calculated with the log-rank test function

To explore whether the epigenetic inactivation of *RSPO2* is an early or a late event in the adenoma to carcinoma sequence, we explored a recent publicly available dataset corresponding to normal colon organoids from FAP patients and control samples [23]. The average DNA methylation status of *RPSO2* in the DMPs identified in our analyses (cg04050867, cg20061155, cg14070647 and cg09970569) in organoids from FAP patients with APC mutations was above the DNA methylation levels observed in control organoids (0.06 vs 0.16) (*p* = 0.06) (Fig. 6A, Additional file 14: Table S10). We further examined the DNA methylation status of *RSPO2* in the TCGA-COAD dataset considering the mutational status of patients with *APC*, *KRAS* and *TP53* mutations (Fig. 6B), which may resemble the temporal acquisition of these mutations in a classical model of the adenoma to carcinoma sequence [24]. It is worth noting that we discarded those patients with mutations in the BRAF gene, which are associated with defective mismatch repair (dMMR) and a serrated adenoma pathway. A significant increase in the DNA methylation levels of the *RSPO2* promoter was observed along the progression of the adenoma to carcinoma sequence (Fig. 6C, Additional file 15: Table S11), and this effect was maximized in the context of patients with *APC/KRAS/TP53* mutations. In addition, a significant anti-correlation between levels of *RSPO2* and the methylation status of its promoter was observed in patients who had all three mutations (Fig. 6D), which is in agreement with the worse overall survival of these patients (Fig. 6E, Additional file 15: Table S11), indicating that the epigenetic repression of *RSPO2* may be associated with the progression of CRC and is more evident at later stages of the adenoma to carcinoma sequence.

## Discussion

In this work, we have carried out a multi-omic analysis to identify alterations in DNA methylation that may act as potential drivers in CRC. In order to identify the most robust epigenetic changes present in independent patient cohorts, we performed the analysis in a local Spanish cohort, as well as in the comprehensive TCGA-COAD cohort, as well as carrying out a dedicated analysis in a set of CRC cell lines from the NCI-60 consortium. Despite the existing limitations in the comparison of primary samples and CRC cell lines [25], this strategy allowed us to delineate those common epigenetic alterations in CRC whose function can be interrogated in a cellular experimental model. Our data revealed that, in the case of DNA hypermethylation, these common regions showed significant enrichments in repressive histone marks such as H3K27me3, along with chromatin states related to bivalent enhancers and Polycomb repressor elements. In the same vein, we also observed significant enrichments in the context of putative binding sites of the Polycomb2-related factor JARID2, confirming the robustness of our observations. These results are consistent with the increase in hypermethylation observed in the context of cancer in CpG islands [26–29], suggesting that DNA methylation at sites decorated by the Polycomb complex in normal cells could play an important role in the tumorigenic process of CRC. On the other hand, we also observed a global DNA hypomethylation scenario in CRC samples compared to their control counterparts, this methylation being mainly enriched in putative binding sites of members of the AP1 complex (FOSB – FRA1). These results recapitulate the observations found in patient samples at the global DNA methylation level [30] and that may be related to the loss of genomic stability observed in cancer [31, 32]. It is worth mentioning that the evaluation of global and intragenic hypomethylation in colorectal adenomas improves patient stratification and colorectal cancer risk prediction [33], indicating the clinical relevance of this regulatory mechanism for the diagnosis and follow-up of CRC patients.

Taking all these observations into account, we performed a more detailed correlation analysis in order to identify candidate genes modulated by strong epigenetic regulation in cancer. We established a strict cut-off in order to identify the most robust changes at the expense of introducing some limitations by losing some relevant changes of lesser magnitude. In addition, we focused on the classical model of epigenetic regulation mediated by the DNA hypermethylation of promoter regions, which has been traditionally associated with the silencing of potential tumour suppressor genes [34, 35]. This strategy allowed us to identify a total of 56 candidate genes which are epigenetically repressed in the tumour context and that could be potentially related to the development of CRC. However, despite the robustness of these correlations, it was still not clear to what extent these candidates could promote tumorigenesis or act as passengers of the tumorigenic process. Therefore, we set out to study the real contribution of these genes in cancer biology by means of a high-throughput interrogation of candidate genomic loci using epigenetic-editing tools based on CRISPR technology [13]. The effectiveness of these tools was in agreement with recent studies [13, 36], indicating that the magnitude of the effect and the range of action of the dCas9-TET1 chimera were within the optimal ranges required for proper epigenetic editing. The induction of DNA hypomethylation in those repressed genes identified in CRC samples revealed a functional role for a number of candidate regions, among them the DNA hypermethylation of *RSPO2* and *GYPC* promoters, which the modulation of exerted a significant effect on the cell viability of DLD1 and HCT116 cell lines. The limited number of significant observations identified in the screen approach may be attributable to the mild reactivation of the gene expression levels obtained through the epigenetic modulation of these genomic loci (~ twofold), as compared to the reactivation of the gene expression obtained with additional CRISPR-dCas9 systems based on transcriptional activation methods (dCas9-VP64). In this regard, it is worth mentioning that more versatile epigenetic-editing platforms that combine the simultaneous modulation of various epigenetic marks, including DNA methylation and different histone activating marks [37], have recently aroused interest and seem to improve the gene reactivation capacity of a certain genomic region of interest, which could represent a more optimal alternative for the interrogation of the functionality of said epigenomic aberrations in cancer. On the other hand, the proposed strategy was focused on the analysis of the cell viability status of CRC cell lines, but we cannot rule out that the epigenetic reactivation of these genes does not have a relevant role in other cellular processes such as cell migration or metastasis, an issue which could be addressed by the use of additional, complementary assays in future studies. It is also worth noting that cancer, unlike more controlled cellular processes such as cell differentiation, shows a high molecular entropy [38], and reversing its phenotype by altering potential tumour suppressor genes one-at-a-time may be a more complex procedure, which is a universal limitation of these experimental screen methodologies.

Despite these limitations, we identified that epigenetic silencing of the *GYPC* and *RSPO2* genes may be involved in the tumorigenic potential of CRC. *GYPC*, also known as Glycophorin C, encodes an integral membrane glycoprotein. Interestingly, recent studies have identified a prognostic potential for this gene in the context of endometrial cancer [39], lung adenocarcinoma [40] and acute myeloid leukaemia [41], where DNA methylation levels are increased in its promoter region, and a decrease in the expression of GYPC is associated with a worse prognosis in these patients. Apart from being highly expressed in red blood cells, the potential mechanisms by which *GYPC* exerts its protective role in these types of tumours remain to be elucidated. On the other hand, the Roof Plate-Specific Spondin-2 (*RSPO2*) gene, which encodes a secreted ligand of leucine-rich repeat-containing G-protein-coupled receptors, plays an important role in Wnt signalling. Previous studies have shown that RSPO2, but not other members of the RSPO protein family, has tumour suppressor activity in colorectal cancer [42]. This inhibition is generated by a non-canonical negative feedback loop of the Wnt/β-catenin signalling a pathway mediated by the interaction of RSPO2 with the G-protein-coupled receptor 5 containing leucine-rich repeats (LGR5), which stabilizes the, membrane-associated zinc and ring finger 3 (ZNRF3) and results in impaired cell proliferation, at least in the context of p53^−/−^ or LGR5-competent CRC cell lines. Our observations indicate that tumour-mediated epigenetic repression and the subsequent epigenetic restoration of *RSPO2* levels affects the cell viability status of CRC cell lines, further supporting previous data generated by Wu and colleagues [42]. The fact that the promoter methylation of this gene also displays a gradual increase along the adenoma to carcinoma sequence further suggests that *RSPO2* may have a functional role as a potential tumour suppressor gene in CRC tumorigenesis.

## Conclusions

In conclusion, our results have explored the potential involvement of DNA methylation as a driving mechanism of CRC and revealed a plausible functional role of epigenetic repression in certain tumour suppressor genes, such as *RSPO2*, in maintaining the viability of tumour cells. These data open the way for the identification of new therapeutic windows based on the epigenetic reactivation of certain tumour suppressor genes that may represent valuable molecular targets for the development of new therapies against CRC.

## Methods

### Acquisition of normal mucosa samples and tumour tissue from CRC patients

The colon samples analysed in this study were collected from the Central University Hospital of Asturias (HUCA). The samples studied comprised 11 healthy colon mucosa and 11 matched tumour tissue from CRC patients. The study was approved by the Clinical Research Ethics Committee of the Principality of Asturias (Ref 116/13) and all the individuals involved provided written informed consent. Additional samples used for validations or downstream computational analyses were obtained from public repositories, specifically the TCGA-COAD consortia (GSE68838) [17], the NCI-60 cell line repository (GSE79185) [18] and a recent study focused on the identification of epigenetic alterations in colon organoids from FAP patients [23].

### DNA methylation analyses

DNA methylation profiling of human control and CRC samples was performed with Illumina’s Infinium HumanMethylation450K BeadChip platform [43]. Bisulphite-only (BS) conversion was performed using the TrueMethyl® protocol for 450 K analysis (version 1.1, CEGX) following the manufacturer’s recommended procedures, and processed DNA samples were hybridized to the BeadChip at the Spanish National Genotyping Center (CEGEN-ISCIII, Madrid, Spain). IDATs were processed using the R/Bioconductor package minfi (v_1.24.0) [44] using the following procedures. Red and green signals from the raw data files were corrected using the ssNOOB algorithm with the default parameters (offset = 15, dyeCorr = TRUE and dyeMethod = “single”) and subsequently normalized using the BMIQ method [45] implemented in the R/Bioconductor package ChAMP (v_2.8.9) [46]. Probes overlapping genetic variants (SNP137Common track from UCSC genome browser), probes located in sexual chromosomes, cross-reactive and multimapping probes and probes with at least one sample with a detection *p* value > 0.01 were discarded for downstream analyses. Technical variability was corrected using the ComBat algorithm from the R/Bioconductor package sva (v_3.26.0). Subsequent β values were extracted with the getBeta minfi function and were used for filtering purposes and for the correlation analysis between DNA methylation and gene expression data. M values were obtained by the logit transformation of the normalized β values with the R/Bioconductor package lumi (v_2.30.0) [47] and were used for statistical purposes assuming homoscedasticity.

A surrogate variable analysis (SVA) [48] was performed to account for possible batch effects or confounding variables using the sva package. Identification of significant DMPs was determined by the moderated *t*-test implemented in the R/Bioconductor package limma (v_3.34.9) [49]. *p* values were corrected for multiple testing using the Benjamini–Hochberg method for controlling the false discovery rate (FDR). An FDR threshold of 0.05, and a minimum absolute difference of 0.25 between mean DNA methylation values of cases and controls was employed to determine DMPs. An additional analysis of differentially methylated regions (DMRs) was performed with the R/Bioconductor package MissMethyl (v_1.12.0) [50]. CpG island methylator phenotype (CIMP) for the different samples was assessed following the criteria used by the TCGA in its molecular classification of CRC samples [17] using a semi-supervised principal component analysis (PCA) with the most variable 1,403 CpG probes present in the Illumina 27 k array platform with the R/CRAN package FactoMineR [51]. The resulting clusters were classified as: CIMPH, CIMPL or No CIMP (combination of the two clusters without CIMP phenotype).

### Region set, chromatin and TFBS enrichment analyses

DMPs were annotated to their corresponding genomic context or genomic location using, respectively, the R/Bioconductor packages IlluminaHumanMethylation450kanno.ilmn12.hg19 (v_0.6.0) and ChIPseeker (v_1.18.0) [52]. Odds ratio (OR) enrichment and statistical significance were calculated by means of two-sided Fisher’s tests. For the different comparisons, appropriate background including all filtered CpG probes interrogated by the Illumina HumanMethylation450K Beadchip platform was used in order to calculate statistical significance.

Chromatin enrichment analyses were performed with the R/Bioconductor package LOLA (v_1.8.0) [53]. DMP enrichments in six histone marks (H3K4me1, H3K4me3, H3K27me3, H3K36me3, H3K9me3 and H3K27ac) were calculated using ChIP-seq tracks from 2 embryonic stem cell- and 5 colorectal-related epigenomes obtained from ENCODE and the NIH Roadmap Epigenome Consortia [20, 21]. Chromatin state data from these same tissue/cell types were obtained from NIH Roadmap’s ChromHMM expanded 18-state model (obtained from http://egg2.wustl.edu/roadmap/). DMP enrichments in TFBSs were performed using data from human meta-clusters obtained from the GTRD database [19]. Clustered peaks corresponding to 476 human TFs across a panel of distinct cells and tissue types were used for statistical purposes. For all enrichment analyses, statistical significance was calculated using one-sided Fisher’s tests (adjusted *p* value < 0.05), comparing the overlap of DMPs with the dataset of interest and using the set of filtered probes from the Illumina HumanMethylation450K platform as background.

### Integration DNA methylation/gene expression ELMER

Correlations between DNA methylation and gene expression data were calculated with the R/Bioconductor package ELMER (v_2.3.7) [54]. Methylation of common DMPs from the HumanMethylationEPIC platform observed in previous analyses was correlated with expression of their most proximal nearby gene to infer functional relationships between the methylation status of a given region and its potential transcriptional targets. Paired DNA methylation loci–gene expression targets were identified using the supervised mode of the get.pair function (permutation size = 2500, Pe = 0.001). Pearson’s correlations were calculated for each significant DNA methylation/gene expression pair and only classical inverse relationships between DNA methylation and gene expression were considered for interpretation purposes.

### Gene ontologies

Gene ontology (GO) analyses were conducted using the GOrilla platform [55]. Significant epigenetically modulated genes were used to interrogate the GOrilla annotation database. The total number of genes identified in the RNA-seq experiment (16,838) was used as the background set for legitimate ontology comparisons.

### Network representation

Network representation between epigenetically regulated genes and their associated DMPs was generated using the R/CRAN package igraph (v_1.2.6). Network nodes represent either correlated CpG sites or genes, while network edges indicate interactions between correlated CpGs-gene pairs.

### gRNA library construction

Guide RNAs targeting gene promoters or DMRs subjected to DNA hypermethylation in CRC were designed using the software CHOPCHOP [56]. At least two different gRNA constructs were designed per target gene and both the coordinates and the efficacy of these sequences are indicated in Additional file 9: Table S8. For gRNA library generation, a pLenti-Guide-Puro- Crispr vector (Origene, #GE100032) was linearized using BamHI and BsmBI restriction endonucleases (NEB, #R0101S and #R0552S respectively) and purified using the NucleoSpin Gel and PCR Clean-up kit (Machery-Nagel, #740609.50). Forward and reverse primers containing the desired gRNA sequence and an additional adaptor sequence were hybridized and subsequently cloned into the linearized vector using the In-Fusion HD cloning kit (Takara, #638911) following the manufacturer’s recommendations. gRNA plasmids were amplified from single colonies using *E. coli* cells (strain DH5α) using a classical Heat Shock method and the resulting constructs were purified using the Qiaprep Spin miniprep kit (Qiagen, #27104).

### Cell line culture and lentiviral production

Human colorectal cancer cell lines DLD1 (#CCL-221) and HCT116 (#CCL-247) were obtained from the American Type Culture Collection (ATCC). Lenti-X™ 293 T Cell Line (#632180) was obtained from Takara. All cells were grown in Dulbecco-modified Eagle medium (DMEM) supplemented with 10% of foetal bovine serum (Sigma-Aldrich, #F6178), 100 U/ml penicillin along with 100 μg/ml streptomycin (Gibco, #15070) and 2.5 g/ml amphotericin B (Gibco, #15290) at 37 °C in a humidified 5% CO_2_ incubator. Viral particles were packaged in Lenti-X™ 293 T cells in the presence of the lentiviral plasmids psPAX2 (Addgene, #12260), MD2.G (Addgene, #12259) and the plasmid of interest, dCas9-TET1 (Addgene, #84475), dCas9-TET1-IM (Addgene, #84479), dCas9-VP64 (Addgene, #61422) or gRNA-specific pLenti-Guide-Puro-Crispr (Origene, #GE100032) using a co-lipofection protocol with Lipfectamine 3000 (Invitrogen, #L3000015). Briefly, a total of 2 × 10^6^ Lenti-X™ 293 T cells were plated in a 25 cm^3^ flask and incubated for 6 h with the lipofection mixture comprising 2.5 μg psPAX2 vector, 1 μg MD2.G vector and 3 μg of the vector of interest. After this incubation time, the culture medium was replaced, and viral particles were collected 24 h later. Either DLD1 or HCT116 cells were co-transduced with the viral-producing medium containing dCas9-TET1, dCas9-TET1-IM, dCas9-VP64 or the gRNAs of interest, and two days after transduction cells were maintained under puromycin selection media.

### Western blotting

Proteins extracts were isolated in RIPA buffer (150 mM NaCl, 1.0% IGEPAL® CA-630, 0.5% sodium deoxycholate, 0.1% SDS, 50 mM Tris, pH 8.0), separated on a 6% SDS-PAGE gel and transferred to an Immobilon-PSQ polyvinylidene difluoride (PVDF) membrane (Millipore, #32031602). The membrane was blocked in phosphate buffered saline buffer with 0.1% Tween 20 (PBS-T) complemented with 10% milk. Detection of protein specific bands was performed using the following primary antibodies (1:1000 dilution in TBS-T with 5% milk): anti-Cas9 (Abcam, #ab191468) and anti- α Tubulin (Abcam, #ab7291). The secondary antibody was a rabbit anti-mouse IgG H&L (Abcam, #ab6728) conjugated to horseradish peroxidase at 1:5000 dilution in TBS-T with 5% milk. Signals were detected using the ECL detection kit (Amersham Biosciences) and Oddysey Fc imaging system.

### DNA extraction and pyrosequencing assays

Genomic DNA was isolated using a standard phenol–chloroform extraction and then subjected to bisulphite conversion using the EZ DNA methylation-gold kit (Zymo Research Corporation). Converted DNA was PCR-amplified using the specific forward- and reverse oligonucleotides listed in Additional file 16: Table S12, and the pyrosequencing reaction was performed using PyroMark Q24 system (Qiagen, Düsseldorf, Germany).

### Cell viability assays

DLD1 and HCT116 cells previously transduced with the appropriate lentiviral constructs were seeded in 96-well plates at a density of 2 × 10^3^ cells in 50 µl of DMEM-FBS and co-transduced with additional 50 μl of 0.45 µm filtered supernatant from the corresponding gRNA Lenti-X™ 293 T transduced cells. Cell viability was interrogated at the indicated timepoint using the CellTiter-Blue Cell Viability Assay kit (Promega, #G808B) according to the manufacturer’s instructions. Fluorescence measurements were obtained using a 530(25) excitation and 590(35) emission filter with the automated microtiter plate reader Synergy HT (BioTek). Well-to-well variability was corrected for using a row-wise normalization approach for proper statistical calculations.

### Screen strategy and data normalization

The high-content cell viability screen to test the functional role of 56 epigenetically repressed genes in CRC was performed in an arrayed format in 96-well plates. The screen strategy was simultaneously tested in DLD1 and HCT116 cells transduced with either dCas9-TET1, dCas9-TET1-IM or dCas9-VP64 constructs, each gene promoter was targeted by two gRNAs (Additional file 9: Table S8) and at least 8 replicates were tested for each condition. At day 0, cells were seeded at a density of 1.5 × 10^3^ cells per well in 50 µl of DMEM-FBS and were co-transduced with an additional 50 μl of 0.45 µm filtered supernatant from the corresponding gRNA mixture or control gRNA. Cell viability was analysed at 120 h after the viral transduction step using the CellTiter-Blue Cell Viability Assay kit (Promega, #G808B) and fluorescence measurements were obtained using an automated microtiter plate reader Synergy HT (BioTek). Subsequent data normalization was performed with the R/Bioconductor package cellHTS2 (v_2.32.0) [57]. Briefly, raw data are subjected to a “by plate” variance normalization using the normalizePlates function on the log-scale data and the median method (scale = “multiplicative”). Then, a batch effect correction of replicates was performed in the scoreReplicates function using the z-score method. Experimental conditions with extreme quantile values (*x* < 0.001 or *x* > 0.999) were discarded from downstream analyses and the ratio between each well and the plate median was calculated. For each of the normalized plates, a scaled, robust z-score for each well was calculated using the average signal of the control condition in each corresponding plate. Scaled Robust z-scores below −1 (average per gene) were considered as statistically significant for downstream purposes.

### RNA extraction and qRT-PCR analyses

RNA extraction from DLD1 and HCT116 cells was performed with the Illustra RNAspin Mini RNA Isolation Kit (Cytiva, # 25050072) according to the manufacturer’s instructions. cDNA was synthesized from total RNA (500 ng) using the SuperScript III Reverse Transcriptase Kit (Invitrogen, #18080044). Quantitative RT-PCR was performed using SYBR Green 2 × PCR Master Mix (Applied Biosystems, #4309155) and the corresponding oligonucleotides are listed in Additional file 16: Table S12. qRT-PCR was carried out using the StepOnePlus real-Time PCR System (Applied Biosystems) and HPRT1 was used as house-keeping gene to standardize data using the ΔΔCt method.

### Survival analyses

Survival analyses were visualized with Kaplan–Meier plots using the resulting sample stratification of TCGA-COAD samples (Stage III and above) in the different mutational categories (APC, APC + KRAS, APC + KRAS + TP53) of the adenoma to carcinoma sequence, and were generated with the R/CRAN packages survival (v.3.2.13) and survminer (v.0.4.9). *p* values indicate significant differences in event rates between the different clusters groups and were calculated using the log-rank test approach.

### Statistical analyses

Statistical analyses were performed using the R programming language (v_3.4.0). Appropriate statistical test were used for the different comparisons performed in this study and the corresponding information is indicated either in the methods section or in the related figure legends.

### Supplementary Information

**Additional file 1: Table S1**. Clinico-biological features of the tumour and control samples used in this study.

**Additional file 2: Table S2**. List of differentially methylated CpGs identified in each of the cohorts used in this study.

**Additional file 3: Table S3**. Statistics related to enrichment analyses of common DMPs at TFBS from the GTRD database.

**Additional file 4: Table S4.** Histone mark enrichment analysis of common DMPs in colorectal-related tissues and embryonic stem cells from the Roadmap and ENCODE consortia.

**Additional file 5: Table S5.** Chromatin state enrichment analysis of common DMPs in colorectal-related tissues and embryonic stem cells from the Roadmap and ENCODE consortia.

**Additional file 6: Table S6.** List of CpG sites displaying robust correlation (>0.5 Pearson corr) between DNA methylation and gene expression in TCGA-COAD as calculated by the R/Bioconductor ELMER package.

**Additional file 7: Table S7.** List of common differentially methylated regions (DMR) between healthy colon samples and the different tumour datasets (E-MTAB-8505, GSE68838 and GSE79185) used in this study.

**Additional file 8: Fig. S1.** dCas9-TET1 modulates the epigenetic status of a conserved cancer-associated DMR. **A** Boxplot illustrating the DNA methylation levels of the indicated significant CpG sites located within the cancer-associated DMRs identified in CRC as determined by the 450 K array platform. Samples are coloured according to their dataset of origin, as indicated in Fig. 1A. **B** Dot plot depicting the DNA methylation status of the CpGs included in the above-mentioned DMR in the context of different dCas9-mediated DNA demethylation or transcriptional reactivation strategies, as determined by a cloning-based bisulphite sequencing protocol. Each line represents a different clone and the genomic coordinates of the different CpG sites contained in this region are indicated at the top. Black circles represent a hypermethylated CpG, while white circles indicate a DNA hypomethylation event. (TIF 2031 KB)

**Additional file 9: Table S8.** List of gRNA sequences designed for the epigenetic modulation of genes with robust correlation between DNA methylation and gene expression in CRC samples.

**Additional file 10: Table S9.** Statistics related to the high-content cell viability screen performed in DLD1 and HCT116 cells transduced with the indicated gRNAs and chimeric constructs.

**Additional file 11: Fig. S2.** Results of the CRISPR-dCas9 demethylation screen strategy in HCT116 cells. **A** Boxplots illustrating the Scaled Robust Z-score data observed for the indicated gene promoters in the context of HCT116 cells transduced with dCas9-TET1 (top), dCas9-TET1-IM (middle) or dCas9-VP64 (bottom) constructs. Genes highlighted in orange reflect those epigenetic modulations that resulted in statistically significant changes in the proliferation rate of HCT116 cells, and control conditions are highlighted in red. **B** Venn diagrams depicting the overlap of significant hits obtained in the different screen strategies performed in HCT116 cells.

**Additional file 12: Fig. S3.** Epigenetic modulation of RSPO2 at its promoter region does not affect the methylation status of intragenic CpG sites. **A** Schema illustrating the genomic position of the RSPO2 gene, the CpG sites analysed in the 450 K methylation platform, the gRNAs designed to modulate the DNA methylation status of its promoter region and the amplicons used for the pyrosequencing assays in the context of the modulated region (Amplicon pyrosequencing A, related to Fig. 5), or a region located downstream of this dCas9-targeted region (Amplicon pyrosequencing B, this figure). **B** Boxplot representing the DNA methylation levels of the indicated significant CpG sites located within the RSPO2 promoter region in CRC samples as determined by the 450 K array platform. Samples are coloured according to their dataset of origin, as indicated in Fig. 1A. **C** Barplots depicting the percentage of DNA methylation observed for the CpG sites included in a location downstream of the modulated differentially methylated region (Amplicon pyrosequencing B) in DLD1 and HCT116 cells in the context of control gRNA (grey) or gRNAs targeting this DMR (blue) in cells transduced with dCas9-TET1- or dCas9-TET1-IM-related chimeras. Data represent mean ± standard deviation of at least 3 independent experiments, and two-sided Welch’s t tests were applied for the different statistical comparisons versus each corresponding control condition. *p value < 0.05; n.s.—nonsignificant.

**Additional file 13: Fig. S4.** Activation of RSPO2 expression by transcriptional mechanisms also impairs the proliferation rate of DLD1 and HCT116 cells. **A** Expression levels of chimeric dCas9-VP64 and β-Tubulin proteins obtained by western blot analyses in control transduced or Cas9 transduced DLD1 and HCT116 cells. The approximate size of the protein products is indicated. **B** Barplots showing RSPO2 gene expression levels observed upon epigenetic modulation of its promoter region in DLD1 (top) and HCT116 (bottom) cells in the context of dCas9-VP64-related chimeras, both in control and RSPO2 targeting RNA conditions. **C** Boxplots representing the normalized cell proliferation rate observed for the indicated gRNA treatments at two different time points (24 and 96 h) in the context of DLD1 and HCT116 cells transduced with dCas9-VP64 constructs. For B, data represent mean ± standard deviation of at least 3 independent experiments, while for C, at least 8 experimental replicas were included. Two-sided Welch’s t tests were applied for the different statistical comparisons versus each corresponding control condition. ***p value < 0.001; **p value < 0.01; *p value < 0.05; n.s.—nonsignificant.

**Additional file 14: Table S10.** Clinico-biological features of organoids from FAP and control patients including the DNA methylation value of the significant probes identified in the context of the RPSO2 gene in this study.

**Additional file 15: Table S11.** Survival data and average DNA methylation/gene expression values of the TCGA-COAD patients included in the analyses related to the adenoma to carcinoma hypothesis.

**Additional file 16: Table S12.** List of primers used in this study.

## Data Availability

Raw Infinium HumanMethylation450K data generated in this study have been deposited in ArrayExpress under the accession number E-MTAB-8505. Raw Infinium HumanMethylation450K data corresponding to additional TCGA-COAD samples, CRC cell lines and colon organoids were obtained from publicly available datasets (GSE68838, GSE79185 and GSE197646, respectively). Paired TCGA-COAD RNA-seq Illumina HiSeq and survival data were obtained from the xenabrowser repository under the TCGA Colon Cancer (COAD) Hub. Processed data and supplemental tables required to reproduce the content of this manuscript have been deposited at Zenodo repository under the accession code https://doi.org/10.5281/zenodo.7761423.

## Abbreviations

CIMP: CpG island methylator phenotype
CRC: Colorectal cancer
CRISPR: Clustered Regularly Interspaced Short Palindromic Repeats
dCas9: Catalytically inactive unit of the Cas9 nuclease
dMMR: Defective mismatch repair
DMP: Differentially methylated probe
DMR: Differentially methylated region
FAP: Familial adenomatous polyposis
FDR: False discovery rate
GO: Gene ontology
GTRD: Gene transcription regulation database
JARID2: Jumonji/ARID Domain-Containing Protein 2
OR: Odds ratio
PCA: Principal component analysis
RSPO2: Roof Plate-Specific Spondin-2
SVA: Surrogate variable analysis
TFBS: Transcription factor binding site
TSS: Transcription start site
UBD: Ubiquitin D
