# Supplementary figures and images for: CRISPR/dCAS9-mediated DNA demethylation screen identifies functional epigenetic determinants of colorectal cancer
Source: Clin Epigenetics. 2023 Aug 24;15:133. doi: 10.1186/s13148-023-01546-1 (PMC10464368; doi:10.1186/s13148-023-01546-1)

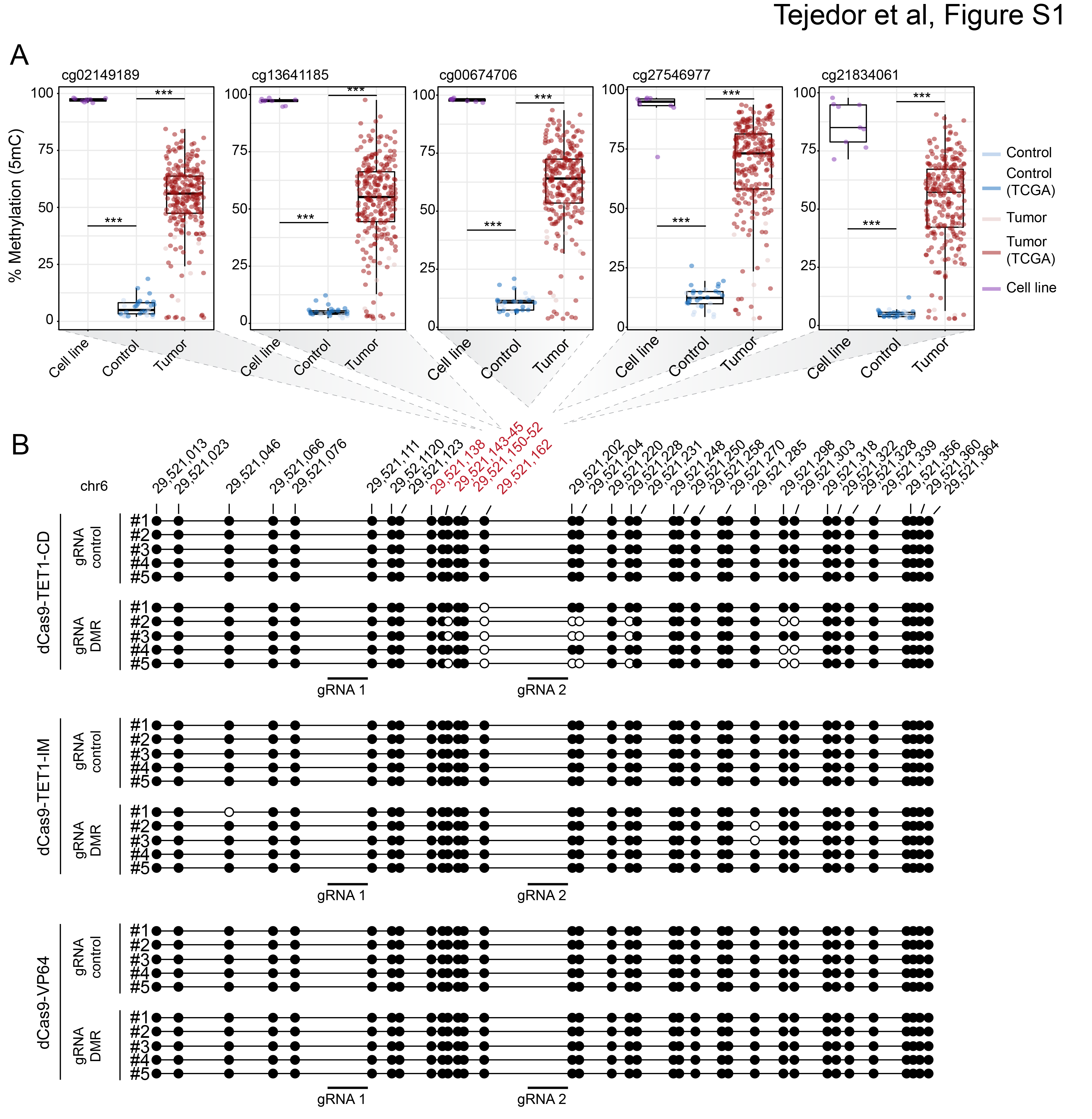

Supplement: Supplementary file 8 — Additional file 8: Fig. S1. dCas9-TET1 modulates the epigenetic status of a conserved cancer-associated DMR. A Boxplot illustrating the DNA methylation levels of the indicated significant CpG sites located within the cancer-associated DMRs identified in CRC as determined by the 450 K array platform. Samples are coloured according to their dataset of origin, as indicated in Fig. 1A. B Dot plot depicting the DNA methylation status of the CpGs included in the above-mentioned DMR in the context of different dCas9-mediated DNA demethylation or transcriptional reactivation strategies, as determined by a cloning-based bisulphite sequencing protocol. Each line represents a different clone and the genomic coordinates of the different CpG sites contained in this region are indicated at the top. Black circles represent a hypermethylated CpG, while white circles indicate a DNA hypomethylation event. (TIF 2031 KB) [file 13148_2023_1546_MOESM8_ESM.tif]

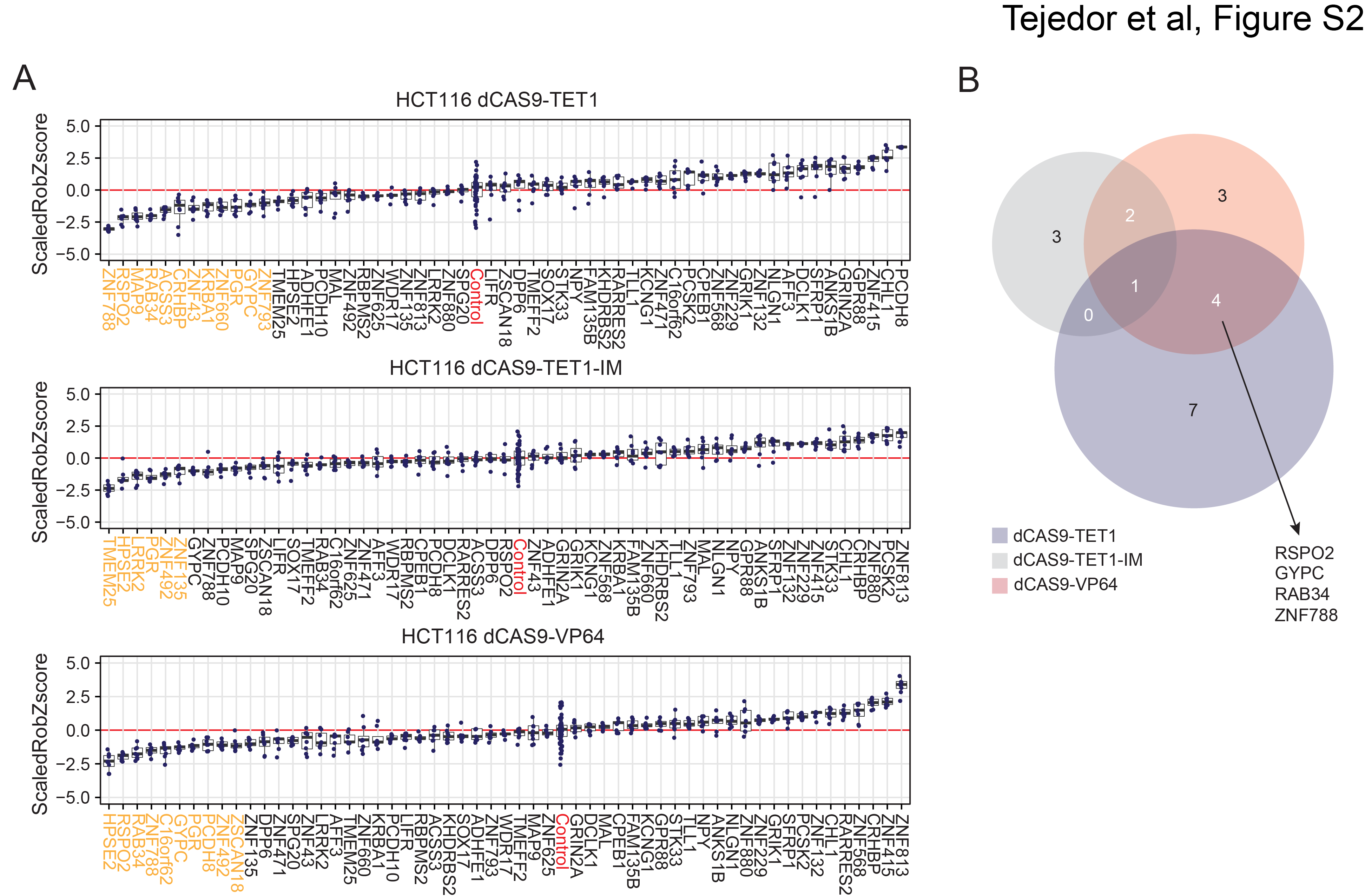

Supplement: Supplementary file 11 — Additional file 11: Fig. S2. Results of the CRISPR-dCas9 demethylation screen strategy in HCT116 cells. A Boxplots illustrating the Scaled Robust Z-score data observed for the indicated gene promoters in the context of HCT116 cells transduced with dCas9-TET1 (top), dCas9-TET1-IM (middle) or dCas9-VP64 (bottom) constructs. Genes highlighted in orange reflect those epigenetic modulations that resulted in statistically significant changes in the proliferation rate of HCT116 cells, and control conditions are highlighted in red. B Venn diagrams depicting the overlap of significant hits obtained in the different screen strategies performed in HCT116 cells. [file 13148_2023_1546_MOESM11_ESM.tif]

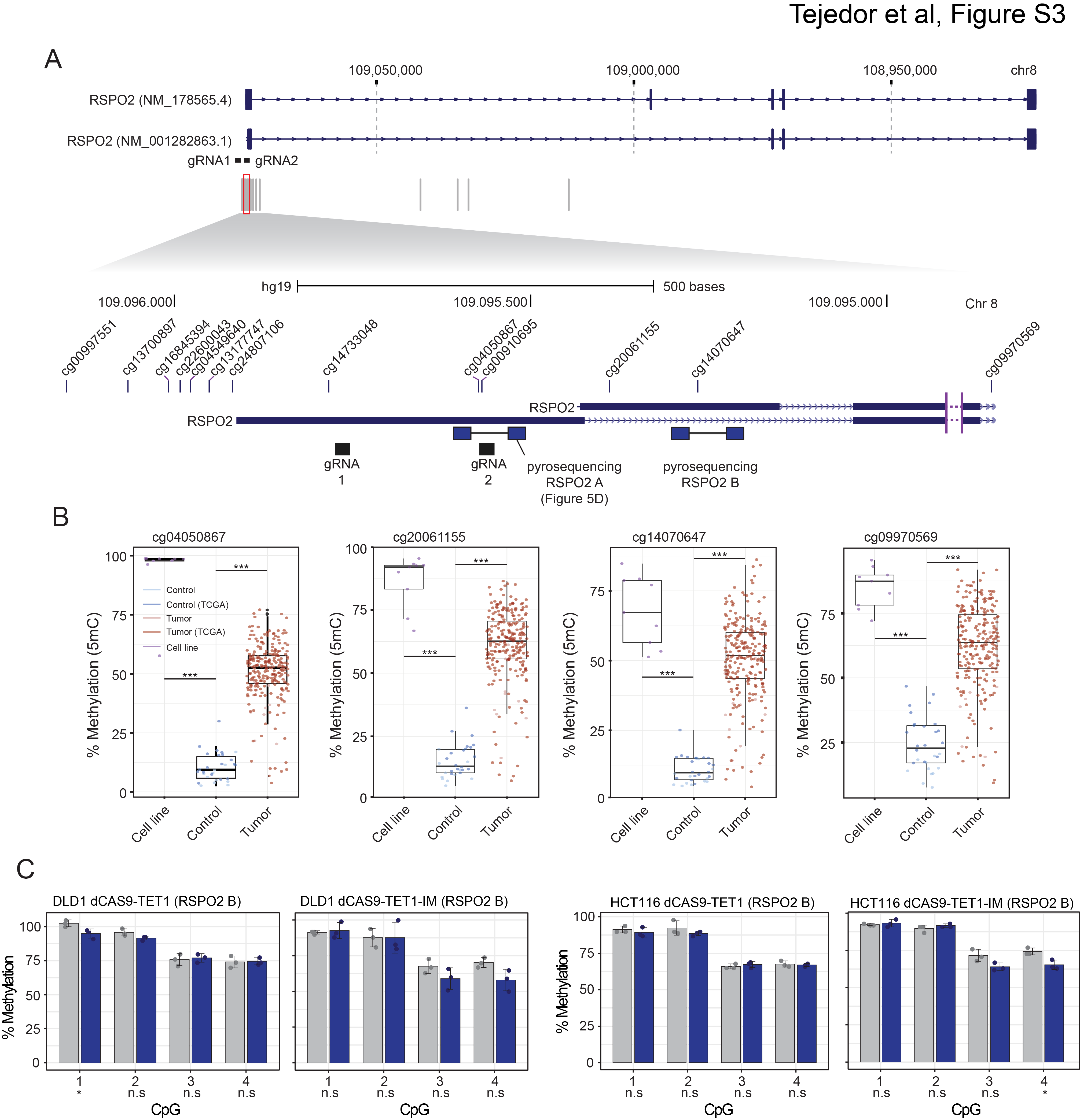

Supplement: Supplementary file 12 — Additional file 12: Fig. S3. Epigenetic modulation of RSPO2 at its promoter region does not affect the methylation status of intragenic CpG sites. A Schema illustrating the genomic position of the RSPO2 gene, the CpG sites analysed in the 450 K methylation platform, the gRNAs designed to modulate the DNA methylation status of its promoter region and the amplicons used for the pyrosequencing assays in the context of the modulated region (Amplicon pyrosequencing A, related to Fig. 5), or a region located downstream of this dCas9-targeted region (Amplicon pyrosequencing B, this figure). B Boxplot representing the DNA methylation levels of the indicated significant CpG sites located within the RSPO2 promoter region in CRC samples as determined by the 450 K array platform. Samples are coloured according to their dataset of origin, as indicated in Fig. 1A. C Barplots depicting the percentage of DNA methylation observed for the CpG sites included in a location downstream of the modulated differentially methylated region (Amplicon pyrosequencing B) in DLD1 and HCT116 cells in the context of control gRNA (grey) or gRNAs targeting this DMR (blue) in cells transduced with dCas9-TET1- or dCas9-TET1-IM-related chimeras. Data represent mean ± standard deviation of at least 3 independent experiments, and two-sided Welch’s t tests were applied for the different statistical comparisons versus each corresponding control condition. *p value < 0.05; n.s.—nonsignificant. [file 13148_2023_1546_MOESM12_ESM.tif]

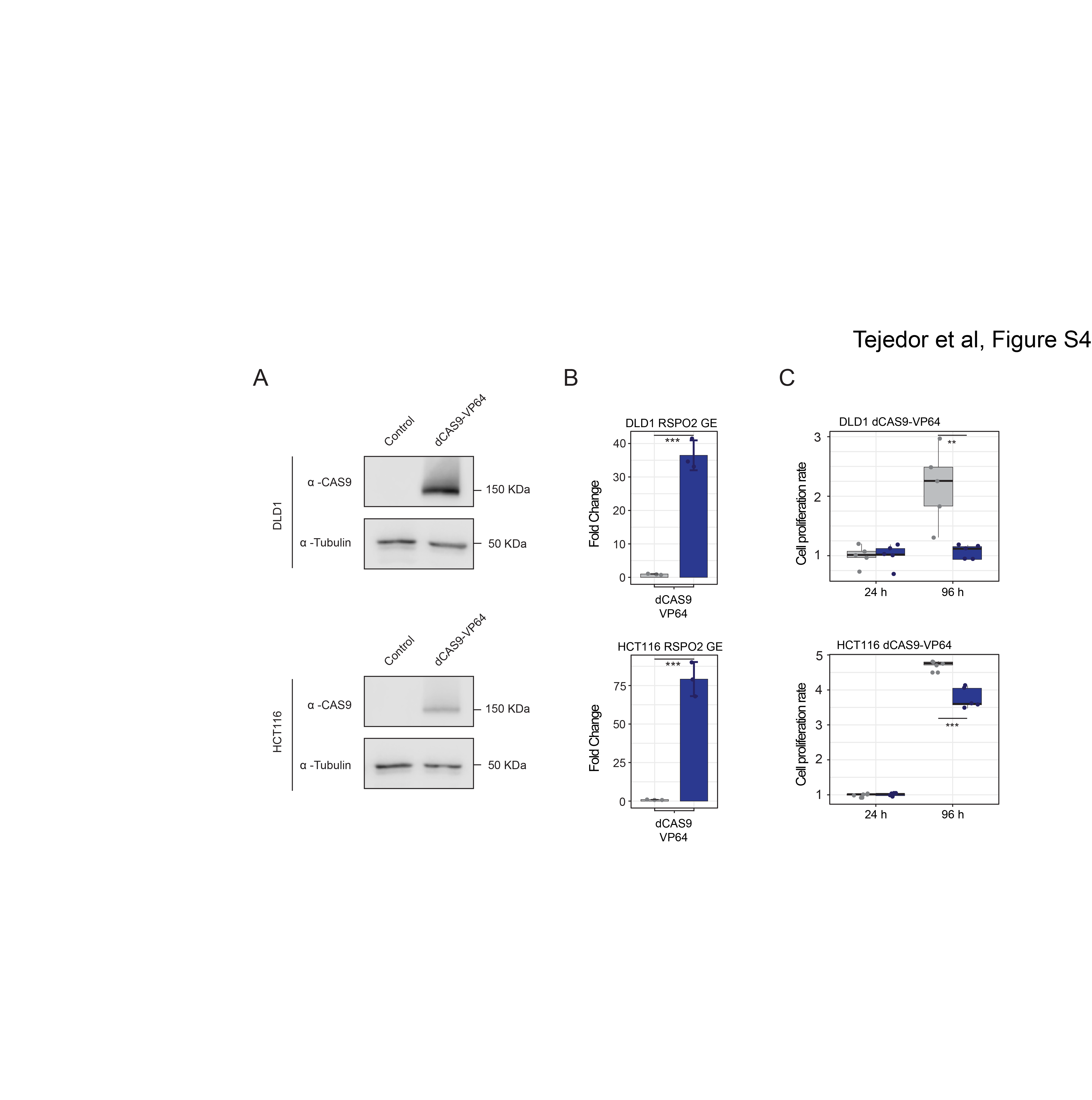

Supplement: Supplementary file 13 — Additional file 13: Fig. S4. Activation of RSPO2 expression by transcriptional mechanisms also impairs the proliferation rate of DLD1 and HCT116 cells. A Expression levels of chimeric dCas9-VP64 and β-Tubulin proteins obtained by western blot analyses in control transduced or Cas9 transduced DLD1 and HCT116 cells. The approximate size of the protein products is indicated. B Barplots showing RSPO2 gene expression levels observed upon epigenetic modulation of its promoter region in DLD1 (top) and HCT116 (bottom) cells in the context of dCas9-VP64-related chimeras, both in control and RSPO2 targeting RNA conditions. C Boxplots representing the normalized cell proliferation rate observed for the indicated gRNA treatments at two different time points (24 and 96 h) in the context of DLD1 and HCT116 cells transduced with dCas9-VP64 constructs. For B, data represent mean ± standard deviation of at least 3 independent experiments, while for C, at least 8 experimental replicas were included. Two-sided Welch’s t tests were applied for the different statistical comparisons versus each corresponding control condition. ***p value < 0.001; **p value < 0.01; *p value < 0.05; n.s.—nonsignificant. [file 13148_2023_1546_MOESM13_ESM.tif]
